# Circulatory miRNAs as Correlates of Elevated Intra-Pancreatic Fat Deposition in a Mixed Ethnic Female Cohort: The TOFI_Asia Study

**DOI:** 10.3390/ijms241814393

**Published:** 2023-09-21

**Authors:** Farha Ramzan, Ivana R. Sequeira-Bisson, Louise W. Lu, Cameron J. Mitchell, Randall F. D’Souza, Mark H. Vickers, Sally D. Poppitt, David Cameron-Smith

**Affiliations:** 1 Liggins Institute, The University of Auckland, Auckland 1023, New Zealanddavid.cameronsmith@unimelb.edu.au (D.C.-S.); 2 The High-Value Nutrition National Science Challenge, Auckland 1023, New Zealands.poppitt@auckland.ac.nz (S.D.P.); 3 Human Nutrition Unit, School of Biological Sciences, Faculty of Science, The University of Auckland, Auckland 1010, New Zealand; 4 The Riddet Institute, Massey University, Palmerston North 4410, New Zealand; 5 School of Kinesiology, The University of British Columbia, Vancouver, BC V6T 1Z1, Canada; 6 School of Medical Sciences, The University of Auckland, Auckland 1023, New Zealand; 7 Department of Medicine, The University of Auckland, Auckland 1023, New Zealand; 8 School of Agriculture, Food and Ecosystem Sciences, The University of Melbourne, Melbourne 3010, Australia

**Keywords:** miRNA, miR-21-3p, miR-320a-5p, ectopic, IPFD, biomarker, liver fat

## Abstract

Ectopic lipid accumulation, including intra-pancreatic fat deposition (IPFD), exacerbates type 2 diabetes risk in susceptible individuals. Dysregulated circulating microRNAs (miRNAs) have been identified as correlating with clinical measures of pancreatitis, pancreatic cancer and type 1 diabetes. The aim of the current study was therefore to examine the association between circulating abundances of candidate miRNAs, IPFD and liver fat deposition as quantified using magnetic resonance imaging (MRI) and spectroscopy (MRS). Asian Chinese (*n* = 34; BMI = 26.7 ± 4.2 kg/m^2^) and European Caucasian (*n* = 34; BMI = 28.0 ± 4.5 kg/m^2^) females from the TOFI_Asia cohort underwent MRI and MRS analysis of pancreas (MR-%IPFD) and liver fat (MR-%liver fat), respectively, to quantify ectopic lipid deposition. Plasma miRNA abundances of a subset of circulatory miRNAs associated with IPFD and liver fat deposition were quantified by qRT-PCR. miR-21-3p and miR-320a-5p correlated with MR-%IPFD, plasma insulin and HOMA2-IR, but not MR-%liver fat. MR-%IPFD remained associated with decreasing miR-21-3p abundance following multivariate regression analysis. miR-21-3p and miR-320a were demonstrated to be negatively correlated with MR-%IPFD, independent of ethnicity. For miR-21-3p, this relationship persists with the inclusion of MR-%liver fat in the model, suggesting the potential for a wider application as a specific circulatory correlate of IPFD.

## 1. Introduction

Ectopic lipid infiltration and accumulation within the visceral organs are key underlying factors for a heightened risk of metabolic disease [1]. Collectively, the actions of pancreatic steatosis and non-alcoholic fatty liver disease (NAFLD) are major determinants of cardiometabolic disease (CMD) [2]. Ectopic lipid accumulation in the pancreas and liver is particularly notable as it has been demonstrated to be a key differentiator for the variation in type 2 diabetes mellitus (T2DM) and CVD risk across differing ethnicities [3].

Whilst considerable attention is directed towards the impact of NAFLD and its relationship to metabolic disease risk [2,4], the importance of intra-pancreatic fat deposition (IPFD) as a causal mechanism implicated in pancreatic β-cell dysfunction and apoptosis has also been established [5,6]. These impairments in pancreatic function are hallmark features for the loss of insulin secretory function present in T2DM [7]. Detailed analysis of IPFD and its relationship to metabolic disease risk has been an ongoing challenge due to the need to accurately quantify lipid accumulation within this small organ located deep in the retroperitoneal abdominal region [8]. Recently described magnetic resonance imaging (MRI) and spectroscopy (MRS) methods are yielding advances in the precision of quantification [9,10]. However, these techniques remain technically challenging, precluding regular application clinically and experimentally [11,12]. Hence, there is a need for suitable informative proxy measures or biomarkers that can be used for preliminary screening or potentially to provide additional insight into the pathogenesis of IPFD.

MicroRNAs (miRNAs) are integral molecular negative regulators of transcriptional processes, with evidence that dysregulated circulatory miRNA abundances contribute to pathophysiological processes associated with the onset of metabolic diseases such as pancreatitis, insulin resistance (IR), liver fat deposition and IPFD [13,14]. miRNA expression is reported to be tissue-specific, although there is evidence for bidirectional crosstalk between metabolically active organs such as the pancreas and liver [15,16]. For example, circulatory miR-375 [17], an islet-cell-enriched miRNA and a critical regulator of β-cell function [18], has been reported to be involved in the development of non-alcoholic liver steatosis [19]. Similarly, miR-122 [20,21], a liver-specific miRNA, has been shown to exhibit significant differences in the blood of severe acute pancreatitis patients compared to healthy controls [22]. Whilst such crosstalk exists between several miRNAs, it is expected that the identification of potential specific biomarkers of miRNAs may help predict or detect the development and progression of steatosis in these organs at an early stage, and therefore allow timely intervention. However, it remains yet to be known if any correlation exists between the abundance of these circulatory miRNAs and the degree of IPFD and liver fat deposition as quantified using MRI/MRS techniques.

The aim of the current study was, therefore, to examine in a cohort of women the correlation between the abundances of a subset of circulatory miRNAs, that have previously been identified as enriched in pancreatic tissue [miR-375, miR-7, miR-361-5p and miR-15a-5p] [23,24], altered in pancreatic disorders such as pancreatitis, insulin resistance (IR) and pancreatic adenocarcinoma [miR-21-3p, miR-126-3p, miR-24-5p, miR-320a] [24,25] or previously reported in circulation as disease markers of these pancreatic disorders [miR-375, miR-7, miR-221 and miR-15a-5p] [25,26]. We analysed the circulatory miRNA levels in the plasma of a cohort of women from the previously described TOFI (Thin on the Outside, Fat on the Inside) cohort consisting of Asian Chinese and European Caucasian ethnicities, lean and overweight, normoglycemic and prediabetic participants [27].

## 2. Results

### 2.1. Characteristics of the Study Population

The clinical and demographic characteristics of the study participants are detailed in Table 1.

Using ethnic-specific cut-off points, 18 women were classified as lean (Asian Chinese (*n* = 10); European Caucasian (*n* = 8)), 26 as overweight, (Asian Chinese (*n* = 11); European Caucasian (*n* = 15)) and 24 obese (Asian Chinese (*n* = 13); European Caucasian (*n* = 11)). Thirteen participants were classified as having pre-diabetes (Asian Chinese (*n* = 6); European Caucasian (*n* = 7)) based on ADA-defined fasting plasma glucose (FPG) concentrations [28].

### 2.2. Quantitative Analysis of the Eleven miRNAs

The abundance of miRNAs across different ethnicities is detailed in Table 2. Additionally, the data showing the abundance of miRNA differences between groups categorized based on BMI (Lean vs. Overweight/Obese) is presented in Supplementary Table S1.

### 2.3. Expression of Circulating miRNAs Correlates with MR-%IPFD but Not with MR-%Liver Fat

**Figure 1:**
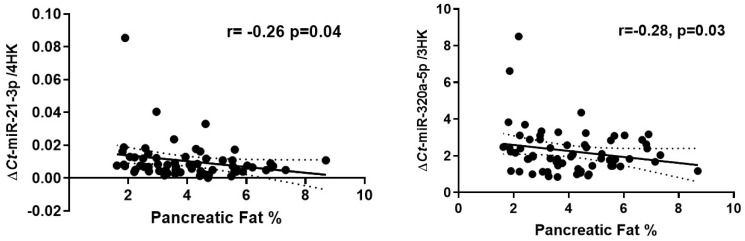
Correlation of circulatory miRNAs with IPFD percentage.

Two of the eleven targeted circulatory miRNAs exhibited significant inverse correlations with MR-%IPFD (miR-21-3p; r = −0.26, *p* = 0.04 and miR-320a-5p; r = −0.28, *p* = 0.03) (Figure 1).

The above figure shows a correlation plots comparing the relative abundance of miR-21-3p and miR-320a-3p to MR-%IPFD. The Pearson’s coefficient is represented by r, and the exact *p*-value is listed. Each point represents an individual sample.

A multivariate linear regression model for miR-21-3p and miR-320a was constructed to establish the association between these miRNAs and MR-%IPFD adjusted for BMI, FPG, plasma insulin and ethnicity (Table 3). The regression model significantly explained the variance in MR-%-IPFD in relation to miRNA expression (*p* < 0.01, R^2^ = 0.43, adj. R^2^ = 0.37).

To ascertain that the abundance of these miRNAs was specific to MR-%IPFD, a multivariate linear regression model for miR-21-3p and miR-320a in relation to MR-%liver fat was constructed. No significant relationship was observed between these miRNA abundances and MR-%liver fat (Table 4).

### 2.4. Expression of Circulating miRNAs Correlates with HOMA2-IR, HbA1c and Fasting Plasma Insulin

**Figure 2:**
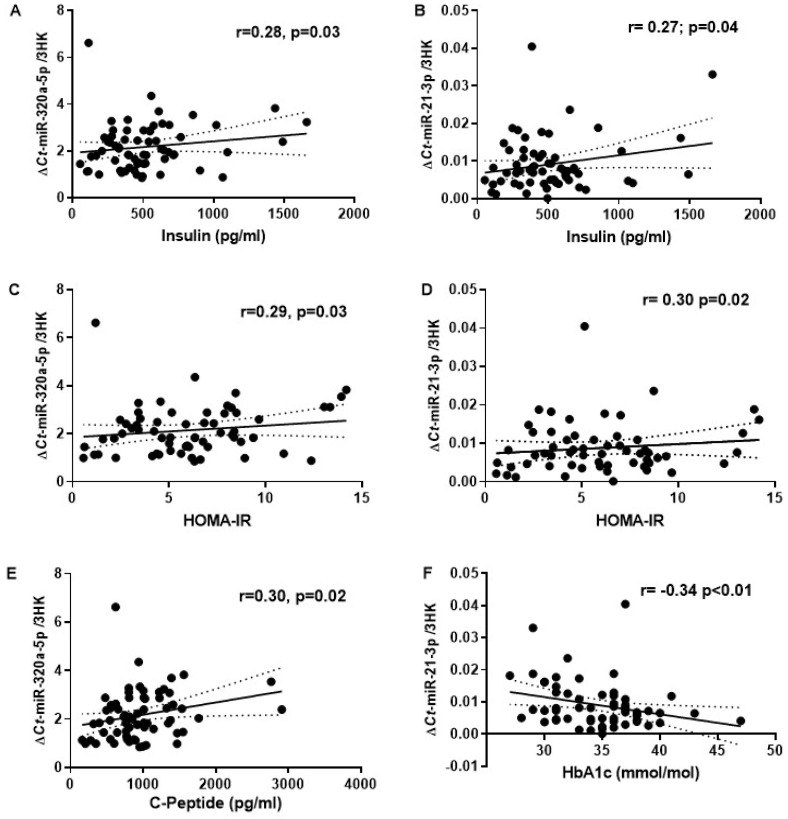
Correlation of circulatory miRNAs with the established T2DM risk markers. (**A**,**B**) Correlation plot comparing the relative abundance of miR-320a-5p and miR-21-3p with plasma insulin. (**C**,**D**) Relative abundance of miR-320a-5p and miR-21-3p with HOMA2-IR. (**E**) Relative abundance of miR-320a-5p with C-Peptide. (**F**) Relative abundance of miR-21-3p and miR-21-3p with HbA1c; r = Pearson’s coefficient with exact *p*-value listed. Each point represents an individual sample.

Additionally, biomarkers of T2DM risk showed a significant correlation with miR-21-3p and miR-320a (Figure 2). Expression of miR-21-3p and miR-320a significantly correlated with fasting plasma insulin (r = 0.27, *p* = 0.04 and r = 0.28, *p* = 0.03, respectively) and HOMA-IR (r = 0.30, *p* = 0.02 and r = 0.29, *p* = 0.03, respectively). Furthermore, miR-320a positively correlated with C-peptide (r = 0.30, *p* = 0.02) while miR-21-3p negatively correlated with HbA1c (r = −0.34, *p* < 0.01). No significant correlation was observed for FPG and circulatory triglycerides with MR-%IPFD.

### 2.5. Receiver Operating Characteristic (ROC) Curve Analysis

**Figure 3:**
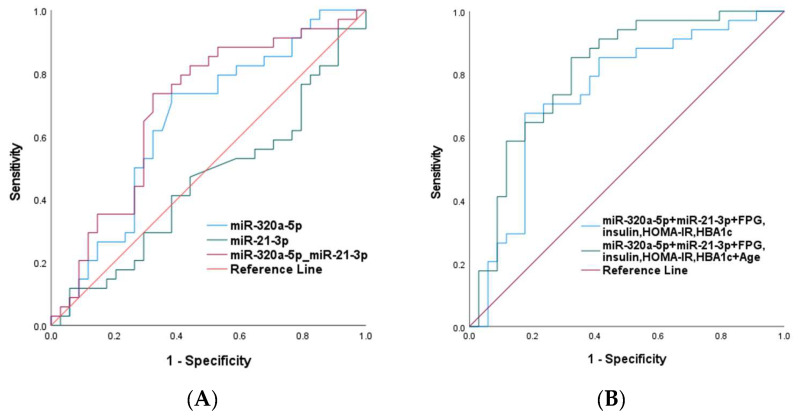
ROC curve analysis using (**A**) miR-320a-5p, miR-21-3p and miR-320a-5p + miR-21-3p (**B**) miR-320a-5p + miR-21-3p + established T2DM risk markers (FPG, insulin, HOMA-IR, HBA1c) and miR-320a-5p + miR-21-3p + established T2DM risk markers + age to evaluate the sensitivity and specificity for predicting IFPD.

The ROC curve and the area under the ROC curve (AUC) were established to evaluate the sensitivity and specificity for predicting IPFD. The accuracy of single miRNAs and the combination of miRNA signatures along with the established T2DM risk markers were assessed. Considered individually, miR-320a-5p showed a statistically significant AUC score, while as miR-21-3p did not reach statistical significance (AUC = 0.647, *p* = 0.037 and AUC = 0.453, *p* = 0.058, respectively). The two miRNAs reached statistical significance when combined together (AUC = 0.690, *p* = 0.007). The association was stronger after adjustment for established T2DM risk markers including FPG, insulin, HBA1c, HOMA-IR as well as after adjusting for age (AUC = 0.74; 95% CI 0.619–0.864; *p* = 0.001 and AUC = 0.806; 95% CI 0.700–0.913; *p* > 0.001, respectively) (Figure 3), in support that the miRNA signature demonstrates good IPFD prediction when used in combination with other established markers.

## 3. Discussion

The circulating abundances of ten candidate miRNAs implicated in the regulation of insulin production and pancreatitis risk were analyzed in relation to MRI-determined IPFD and MRS-determined liver fat percentage. Of these miRNAs, miR-21-3p and miR-320a exhibited a negative correlation, specifically for MR-%IPFD. Furthermore, the abundances of these miRNAs correlated with biomarkers of metabolic disease, which includes circulatory C-peptide, HOMA2-IR, plasma insulin and HbA1c. Moreover, in a multivariate linear regression model involving BMI, FPG and plasma insulin as covariates, miR-21-3p showed a significant negative association with IPFD irrespective of ethnicity [29,30].

Upregulated expression of miR-21-3p is reported to be a general feature of tissue inflammation and fibrosis [31]. miR-21-3p is shown to have pro-adipogenic characteristics [32], with an upregulated expression in subcutaneous adipose tissues of obese human and animal models [33]. Interestingly, under normal physiological conditions, miR-21-3p is highly expressed in the hepatocytes of mice, although minimally active [34]. However, upon being challenged with a high-fat diet, miR-21-3p promotes metabolic derangements, including glucose intolerance, IR and fat deposition in mice [35]. Furthermore, ref. [31] in human models of acute pancreatitis, a reduced abundance of miR-21 has been observed. In line with the literature, this study also observed a negative association between the measured circulatory abundance of miR-21-3p and that of MR-%IPFD. Since IPFD and pancreatitis are closely associated, the observed negative abundance of miR-21 could possibly be an early indicator of later-life pancreatitis [36,37]. However, to confirm these results, there is an important need to confirm the functionality of these results in animal and cell models.

Although little is known about the role of miR-320a and IPFD, altered expression of miR-320a has been observed in pancreatic cancer, IR and pancreatic fibrosis [26,38]. Considering that IPFD is associated with chronic inflammation and various other conditions, including acute and chronic pancreatitis, T2DM and pancreatic cancer, the identified correlation between miR-320a and IPFD could potentially serve as an indicator of the risk factors for these pancreatic diseases [6]. Clearly, mechanistic analysis is required to confirm the function of these miRNAs in the complex regulation of pancreatic function.

Variation in the deposition of intra-pancreatic fat has been reported in different ethnicities [39], with additional evidence for ethnicity impacting the expression patterns of miRNAs [40]. Therefore, to elucidate the effect of different ethnicities of our study participants on the expression of miR-21-3p and miR-320a, a partial correlation of these miRNAs with MR-%IPFD, while controlling for ethnicity, was performed. Interestingly, the expression of these miRNAs still showed an inverse correlation with the MR-%IPFD, thus suggesting that miR-21-3p and miR-320a are associated with IPFD independent of ethnicity.

There are some limitations to the findings of this study. There has been evidence of differences in the physiological processes of the pancreas based on the sex of an individual. For example, while analyzing the secretin-induced exocrine pancreatic response in healthy men and women above and below the age of 45, it was observed that women over the age limit, compared to the males, secreted significantly less water and bicarbonate [41]. Moreover, the authors also observed age- and sex-dependent variations for lipase secretion, with a decreased secretion in women per mg of pancreatic tissue [41]. There is no consensus around sex-specific miRNA expression and IPFD. The present study was undertaken in females only and further studies examining potential sex-specificity in circulatory miRNA profiles and their associations with IPFD are required. In addition, plasma miRNA profiling provides limited insights into tissue-specific exosomal miRNA expression as it cannot conclusively identify the tissue source of these miRNAs [42]. Therefore, further elucidation of the clinical implications of the altered miRNA abundances on IPFD and other metabolically active organs is required to ascertain systemic effects.

## 4. Methods and Materials

### 4.1. Study Design

The plasma samples utilized in this study were archived from the previously conducted TOFI cohort study [27], with ethical approval inclusive of these secondary analyses from the Health and Disabilities Ethics Committee (HDEC) Auckland, New Zealand (16/STH/23). The study was conducted according to the Declaration of Helsinki guidelines. The study is registered with the Australian New Zealand Clinical Trials Registry (ACTRN12616000362493).

### 4.2. Study Population

Analysis was performed on 68 women (34 Asian Chinese and 34 European Caucasian) who underwent extensive MRI and MRS analyses as previously described [27]. The women were aged between 20 and 70 years [27]. Women were normoglycemic or had impaired fasting glucose (IFG), as defined by the American Diabetes Association [28], self-reported healthy with no significant disease, no significant weight gain or loss (>10%) in previous 3 months, and no contraindications for MRI/S procedures were included.

### 4.3. Sample Collection

Fasting blood samples were collected from each participant in EDTA-coated tubes and immediately centrifuged at 1300× *g* for 10 min at 4 °C for plasma separation. The resultant plasma was aliquoted and stored at −80 °C until further analysis.

### 4.4. Anthropometric and Biochemical Analysis

Height, weight, waist circumference and blood pressure were measured at fasting, as previously reported [43]. Body mass index (BMI) cut-off points were determined as Asian Chinese; overweight ≥ 24 kg/m^2^, obese ≥ 28 kg/m^2^; European Caucasian: overweight ≥ 25 kg/m^2^, obese ≥ 30 kg/m^2^. Biochemical measures were assessed as described previously [27]. Among these measurements, glucose was analyzed by the hexokinase method, total cholesterol and high-density lipoprotein-cholesterol (HDL-C) were measured using the cholesterol-esterase/cholesterol oxidase/peroxidase method, and low-density lipoprotein-cholesterol (LDL-C) was calculated using the Friedwald formula [27]. Triglycerides (TAGs) were analyzed by the lipase/glycerol kinase method [27]. Plasma insulin (limit of detection (LOD): 87 pg/mL) and C-peptide (LOD: 9.5 pg/mL) were measured by multiplex immunoassay (MILLIPLEX^®^MAP Human Metabolic Hormone Magnetic Bead Panel, Darmstadt, Germany). Glycosylated hemoglobin (HbA1c) was determined by capillary electrophoresis (CAP2FP, Sebia, France). Homeostasis model assessment of IR (HOMA2-IR) was determined online using the HOMA2 Calculator© (The University of Oxford 2013, Version 2.2.3) [44].

### 4.5. Pancreatic and Liver Fat Content Quantification by Magnetic Resonance Imaging (MRI) and Spectroscopy (MRS)

Intra-pancreatic fat percentage was quantified using a 3T Magnetom Skyra scanner, VE 11A (Siemens, Munich, Germany), using a T1-weighted, 3D dual gradient echo sequence (VIBE) 2-point Dixon method as previously described [12]. Two candidate pancreas (5 mm each) fat fraction (FF) maps with a visible head, body and tail were created using MRI images. To estimate MR-%IPFD, three regions of interest (ROI) were placed in each image’s head, body, and tail, respectively. Furthermore, a 1–20% threshold was applied to eliminate the potential inclusion of non-parenchymal tissue within the selected ROI. The average fat of both candidate pancreas FF images was utilized to calculate MR-%IPFD.

Liver fat content was measured using the MRS method as described previously [12]. Briefly, a 2 × 2 × 2 cm^3^ voxel was placed in the right lobe avoiding blood vessels and the biliary tree; spectra were obtained in transverse, coronal, and sagittal planes ± water suppression. Liver fat was expressed as % calculated *v*/*v* of fat and water.

### 4.6. Circulating Total RNA Extraction

Plasma (250 μL) was used for total RNA (including miRNAs) isolation as described previously by D’Souza et al. [45].

### 4.7. cDNA Synthesis

A fixed volume of 2 μL of total RNA was used as input for each cDNA synthesis reaction using TaqMan™ Advanced miRNA cDNA Synthesis Kit (Catalogue number: A28007, Applied Biosystems, Foster City, CA, USA) following the manufacturer’s recommendations [46].

### 4.8. Circulating miRNA Real-Time Quantitative PCR (qPCR)

A total of eleven [miR-375, miR-7, miR-361-5p and miR-15a-5p, miR-21-3p, miR-126-3p, miR-146-5p, miR-24-5p, miR-320a, miR-221, and miR-17-5p] custom human miRNA assays (TaqMan miRNA Assays, Applied Biosystems) were utilized. According to the manufacturer’s instructions, a PCR reaction mixture of Master Mix and PCR primer (TaqMan^®^ Fast Advanced Master Mix and TaqMan miRNA Assays) was prepared, and amplification was performed on a Quant Studio™ 6 Flex Real-Time PCR System (Applied Biosystems) using cycling parameters recommended by Applied Biosystems. Samples with *CT* > 35 were excluded from the analysis.

Two technical replicates were used for each sample. Hemolysis of samples was monitored by comparing miR-23a-3p expression, reported to be unaffected by hemolysis, with miR-451a, a highly expressed miRNA in red blood cells, ΔCt (miR-23a-3p-miR-451a), with a ΔCt of >7 indicating a high risk of hemolysis [47]. For quality control of cDNA synthesis, an exogenous spike-in (cel-miR-238) was spiked in all samples. miRNA expression data were normalized using a geometric mean of two endogenous miRNAs, miR-423-5p (Asian Chinese; Ct = 22.14 ± 2.15, European Caucasian; Ct = 21.89 ± 2.58 (*p* = 0.66)) and miR-191-5p (Asian Chinese; Ct = 24.90 ± 1.91, European Caucasian; Ct = 23.87 ± 2.30 (*p* = 0.11)) along with an exogenous spike-in cel-miR-238 (Asian Chinese; Ct = 20.95 ± 0.20, European Caucasian; Ct = 20.48 ± 0.25 (*p* = 0.13)). The abundance of miRNAs was measured using the 2^(−ΔCt)^ method [48].

### 4.9. Statistical Analysis

Data were expressed as mean ± standard deviation (SD). Pearson’s correlation coefficients assessed the association between circulatory miRNA expression, MR-%IPFD and MR-%liver fat. miRNAs that showed a significant correlation (*p* ≤ 0.05) with MR-%IPFD were further used to construct a multivariate linear regression model. Regression models were adjusted for ethnicity and biochemical and physiological parameters, including BMI, FPG and plasma insulin. The standard error (SE) was used to estimate the intercept and slope of linear regression equations, and analysis of variance (ANOVA) was used to demonstrate the significance of the whole model with statistical significance set at *p* ≤ 0.05. Receiver operator characteristic (ROC) curves and area under the curve (AUC) were established to evaluate the sensitivity and specificity of the circulatory miRNAs for predicting IPFD. All data were analyzed using SPSS 25.0 (SPSS Inc., Chicago, IL, USA), and all graphs were prepared using GraphPad Prism-9 (version 3.1) (GraphPad Software, Boston, MA, USA).

## 5. Conclusions

This study showed a correlation between MR-%IPFD and the abundance of circulating miRNAs. Irrespective of ethnicity, miR-21-3p was negatively correlated with the MR-%IPFD but not with MR-%liver fat. A significant correlation of both miRNAs was also observed with biomarkers related to impaired pancreatic endocrine function, including HOMA2-IR, HbA1c, and fasting plasma insulin. These analyses were exploratory and preliminary. Therefore, the clinical utility of these miRNAs as biomarkers of organ steatosis remains uncertain. However, this study highlights important observations that would help bridge the gap in understanding the underlying mechanisms linking miRNAs and the pathophysiology of IPFD and liver fat deposition.

## Figures and Tables

**Table 1:** The clinical and demographic characteristics of the study participants.

Participant Characteristics	Asian Chinese (*n* = 34)	European Caucasian (*n* = 34)	*p*-Value
Age (years)	41.0 ± 13.0	47.8 ± 15.4	0.05
BMI (kg/m^2^)	26.7 ± 4.2	28.0 ± 4.5	0.24
Waist Circumference (cm)	85.6 ± 11.1	91.7± 13.9	0.05
BP-Systolic (mmHg)	120 ± 22	120 ± 19	0.93
BP-Diastolic (mmHg)	65 ± 12	64 ± 8	0.61
HbA1c (mmol/mol)	35.5 ± 3.8	33.9 ± 3.9	0.09
Fasting Plasma Glucose (FPG) (mmol/L)	5.2 ± 0.5	5.1 ± 0.7	0.52
Total Cholesterol (mmol/L)	4.5 ± 0.9	5.2 ± 0.9	0.004
LDL-C (mmol/L)	2.5 ± 0.7	2.9 ± 0.9	0.02
HDL-C (mmol/L)	1.4 ± 0.4	1.8 ± 0.4	0.001
Triglycerides (mmol/L)	1.3 ± 0.7	1.0 ± 0.5	0.09
HOMA2-IR	1.8 ± 1.0	1.6 ± 1.2	0.37
MR-%IPFD	4.3 ± 2.0	4.1 ± 1.9	0.69
MR-%liver fat	4.6 ± 4.7	3.7 ± 4.8	0.47

Data are expressed as Mean ± SD. BMI, body mass index; BP, blood pressure; HDL-C, high-density lipoprotein-cholesterol; LDL-C, low-density lipoprotein-cholesterol; HOMA2-IR, homeostasis model assessment of insulin resistance; HbA1c, glycosylated hemoglobin; MR, magnetic resonance; IPFD, Intra pancreatic fat deposition.

**Table 2:** The abundance of miRNAs across different ethnicities.

miRNA Abundance	Asian Chinese (*n* = 34)	European Caucasian (*n* = 34)	*p*-Value
miR-24-5p	0.77 ± 0.52	0.26 ± 0.02	0.20
miR-17-5p	0.37 ± 0.04	0.75 ± 0.05	<0.01
miR-221-3p	0.46 ± 0.03	0.54 ± 0.04	0.22
miR-15a-5p	0.29 ± 0.02	0.44 ± 0.04	0.01
miR-361-5p	1.13 ± 1.08	0.04 ± 0.004	0.19
miR-21-3p	0.007 ± 0.001	0.005 ± 0.001	0.26
miR-7-5p	0.0009 ± 0.000	0.001 ± 0.0002	0.25
miR-320a-3p	2.39 ± 0.23	1.76 ± 0.17	0.03
miR-146-5p	0.05 ± 0.01	0.04 ± 0.00	0.25
miR-126-3p	0.75 ± 0.14	1.11 ± 0.09	0.03
miR-375-5p	0.19 ± 0.18	0.03 ± 0.05	0.22

Data are expressed as Mean ± SD. miR; microRNA.

**Table 3:** Multivariate linear regression model showing association of MR-%IPFD with alterations in the circulatory miR-21-3p and miR-320a-5p expression.

Model	B	SEM	Expected (B)	*p*-Value
(Constant)	−0.29	0.19	−1.48	0.14
miR-21-3p	−3.87	1.63	−2.37	0.02
miR-320a-5p	−0.02	0.01	−1.53	0.13
BMI (kg/m^2^)	0.01	0.00	3.07	0.00
FPG (mmol/L)	0.11	0.03	3.60	0.00
Insulin (pg/mL)	−0.00	0.00	−0.48	0.62
Ethnicity	0.00	0.03	0.23	0.81

The model was fitted for BMI (body mass index), FPG (fasting plasma glucose), plasma insulin and ethnicity. The regression β coefficient is represented by B.

**Table 4:** Multivariate linear regression model showing association of MR-%liver fat with alterations in the circulatory miR-21-3p and miR-320a-5p expression.

Model	B	SEM	Expected (B)	*p*-Value
(Constant)	−1.04	0.47	−2.21	0.03
miR-21-3p	−4.92	3.82	−1.28	0.20
miR-320a-5p	−0.02	0.04	−0.50	0.61
BMI (kg/m^2^)	0.02	0.01	2.16	0.03
FPG (mmol/L)	0.12	0.08	1.52	0.13
Insulin (pg/mL)	0.00	0.00	1.86	0.06
Ethnicity	0.05	0.09	0.56	0.57

The model was fitted for BMI (body mass index), FPG (fasting plasma glucose), plasma insulin and ethnicity. The regression β coefficient is represented by B.

## Data Availability

The study is registered with the Australian New Zealand Clinical Trials Registry at anzctr.org.au (ACTRN12616000362493). The datasets generated for this study are available on request to the corresponding author.

## Supplementary Materials

The following supporting information can be downloaded at: https://www.mdpi.com/article/10.3390/ijms241814393/s1.
