# Supplementary material for: Circulatory miRNAs as Correlates of Elevated Intra-Pancreatic Fat Deposition in a Mixed Ethnic Female Cohort: The TOFI_Asia Study
Source: Int J Mol Sci. 2023 Sep 21;24(18):14393. doi: 10.3390/ijms241814393 (PMC10532072; doi:10.3390/ijms241814393)
Supplement: Supplementary file 1 [file ijms-24-14393-s001.zip › ijms-2572721-supplementary.pdf]

miRNA Abundance	Lean (18-25 kg/m^2) (n=18)	Overweight/Obese(>25 kg/m^2) (n=50)	p-value
**miR-24-5p**	0.28 ± 0.52	0.32± 0.02	0.48
**miR-17-5p**	0.64 ± 0.04	0.60 ± 0.05	0.72
**miR-221-3p**	0.51 ± 0.03	0.55± 0.04	0.46
**miR-15a-5p**	0.45 ± 0.02	0.37 ± 0.04	0.30
**miR-361-5p**	0.05± 1.08	0.05 ± 0.004	0.44
**miR-21-3p**	0.008±0.001	0.008±0.001	0.87
**miR-7-5p**	0.001 ± 0.000	0.001 ± 0.0002	0.69
**miR-320a-3p**	1.96 ± 0.23	2.07 ±0.17	0.92
**miR-146-5p**	0.05 ± 0.01	0.05 ± 0.00	0.92
**miR-126-3p**	1.23± 0.14	1.03 ± 0.09	0.37
**miR-375-5p**	0.06 ± 0.18	0.06 ± 0.05	0.90

Supplementary Table S1: Quantitative analysis of the Eleven miRNAs

The abundance of miRNAs between groups categorized based on BMI (Lean Vs Overweight/Obese)
Data are expressed as Mean ± SEM. miR; microRNA.
